# Reward-Based Eating Drive Is Associated with Distinct Resting-State Connectivity Patterns: A RED-13 and fMRI Study

**DOI:** 10.3390/nu18213480

**Published:** 2026-10-23

**Authors:** Dana Mihaela Ciobanu, Anca-Elena Crăciun, Adriana Rusu, Ruben Emanuel Nechifor, Nicu Cătălin Drăghici, Andreia Mocan, Cornelia Bala

**Affiliations:** 1 Department of Diabetes and Nutrition Diseases, Faculty of Medicine, “Iuliu Hațieganu” University of Medicine and Pharmacy, 2–4 Clinicilor Street, 400006 Cluj-Napoca, Romania; dana.ciobanu@umfcluj.ro (D.M.C.); anca.craciun@umfcluj.ro (A.-E.C.); cbala@umfcluj.ro (C.B.); 2 Center for Diabetes and Nutrition Diseases, Emergency Clinical County Hospital Cluj, 400006 Cluj-Napoca, Romania; andreiamocan@gmail.com; 3 2nd Department, Faculty of Nursing and Health Sciences, “Iuliu Hațieganu” University of Medicine and Pharmacy, 4 Louis Pasteur Street, 400349 Cluj-Napoca, Romania; 4 International Institute for the Advanced Studies of Psychotherapy and Applied Mental Health Department of Clinical Psychology, Psychotherapy Babes-Bolyai University, 400294 Cluj-Napoca, Romania; ruben.nechifor@ubbcluj.ro; 5 Center of Excellence for Neuroscience and Mental Health, Faculty of Psychology and Sciences of Education, Babes-Bolyai University, 400031 Cluj-Napoca, Romania; 6 INSPIRE Platform, Babes-Bolyai University, 400031 Cluj-Napoca, Romania; 7 Department of Clinical Neurosciences, “Iuliu Hațieganu” University of Medicine and Pharmacy, 400012 Cluj-Napoca, Romania; nicu.draghici@umfcluj.ro; 8 “IMOGEN” Institute, Centre of Advanced Research Studies, Emergency Clinical County Hospital Cluj, 400012 Cluj-Napoca, Romania

**Keywords:** RED-13, reward-based eating, food appetite, resting state fMRI, regional homogeneity, cerebellum

## Abstract

**Background/Objectives**: Eating for pleasure rather than hunger is widespread in the modern food environment. The Reward-Based Eating Drive Scale (RED-13) captures three dimensions of reward-related eating: lack of satiety, loss of control over eating, and preoccupation with food. This cross-sectional study aimed to evaluate the relationship between resting-state functional magnetic resonance imaging (fMRI) metrics and the RED-13 subscales. **Methods:** RED-13 scores, subjective food appetite, and resting-state brain function were measured in 99 participants between March 2022 and May 2023. Brain function was assessed using degree centrality (DC), regional homogeneity (ReHo), and voxel-mirrored homotopic connectivity (VMHC). **Results:** Loss of control over eating and preoccupation with food positively correlated with appetite for high fat, high sweet, and low sweet foods. Loss of control over eating positively correlated with DC in the left cerebellar lobule IX (*r* = 0.416), ReHo in the left brainstem (*r* = 0.399), and VMHC in the right and left brainstem (*r* = 0.432) and negatively with ReHo in the right calcarine sulcus (*r* = −0.434). The lack of satiety subscale positively correlated with ReHo in the left superior frontal gyrus (*r* = 0.389). The preoccupation with food subscale positively correlated with DC and ReHo in the right middle frontal gyrus. **Conclusions:** Reward-based eating measured by RED-13 is associated with distinct patterns in the resting-state fMRI metrics within reward processing and cognitive control. These neural correlates, supported by the association of RED-13 with appetite, are consistent with the hypothesis that RED-13 captures a trait-like disposition toward reward-driven eating that might be reflected in the resting-state brain activity.

## 1. Introduction

Energy intake in humans is governed by two different control systems. The homeostatic mechanism responds to metabolic need—caloric and other metabolic deficits—and is organized around the hypothalamus, which integrates peripheral hormonal signals and vagal afferent nerve input to generate hunger and satiety sensations. The non-homeostatic process is the second mechanism involved in food intake and it is driven by cognitive, emotional and environmental factors [1].

Hedonic feeding is a consequence of the overriding of the inhibitory mechanisms that regulate caloric intake, through activation of corticolimbic reward circuitry. In environments where highly palatable foods are continuously available, reward dominates decisions regarding the type and quantity of food to be eaten, even in the absence, or in opposition to, metabolic requirements [2]. Eating for pleasure rather than in response to hunger is widespread in the modern food environment in developed societies, where easy access to highly palatable foods, especially those rich in fats, sugar, and salt, constantly draw individuals toward a rewarding eating experience rather than the covering of homeostatic caloric need [3]. Over time, such exposure has been proposed to alter the brain’s reward circuitry, potentially driving addiction-like behavior and overeating [4] and increasing the obesity risk. Both positive and negative emotions can motivate reward-related eating, contributing respectively to positive and negative reinforcement of the emotional state [5].

Data supporting the hedonic system involvement in motivation and reward come from functional magnetic resonance imaging (fMRI) studies reporting increased responses to visual food cues in people with obesity [6,7] or across weight status [8]. However, recent evidence suggests less consistent weight-related group differences [9] and points to a state-dependent response rather than to a stable organization of the reward system [10]. Resting state fMRI which measures task-independent low-frequency blood-oxygenation-level-dependent (BOLD) signals has been used to assess the intrinsic interaction between brain regions [11]. Several voxel-wise metrics derived from these signals are able to describe complementary properties of that organization: regional homogeneity (ReHo), which indexes the local synchronization of a voxel with its immediate neighbors; degree centrality (DC), which quantifies the number of functional connections a voxel maintains across the whole brain; and voxel-mirrored homotopic connectivity (VMHC), which reflects the coordination between symmetrical regions of the two hemispheres [12]. These metrics have been reported to be altered in obesity [13,14] and in relation to self-reported eating traits such as food craving [6,7].

Reward eating operationalized by the Reward-Based Eating Drive Scale (RED) is a self-report instrument designed to evaluate three dimensions of reward-related eating: lack of satiety, loss of control over eating, and preoccupation with food [15]. This scale was broadened to 13 items in order to extend coverage across the spectrum of reward-related eating (RED-13) [16]. Multiple studies have supported its robustness across several populations [17,18], with or without obesity [19], though its scalar variance across countries has not been established. Higher RED-13 scores have been shown to indicate a greater tendency toward reward-based eating behaviors such as emotional eating, food cravings, and reward-seeking [17]; its relationship with the brain reward circuitry has so far only been postulated. Rather than being a transient state, the RED-13 was designed to detect a stable tendency toward reward-driven eating [15,16]. On the other hand, momentary appetite is a condition that is typically measured using visual analog scales that have been approved for the evaluation of appetite sensations [20]. Both the convergence of the two measures and the reflection of the trait in intrinsic brain organization bear on this interpretation [6].

In this context, we hypothesize that reward-based eating drive is associated with altered intrinsic brain activity, particularly in regions involved in reward processing and in the regulatory control of eating. In our cross-sectional study, we aimed to evaluate the relationship between the resting-state metrics and the RED-13 subscale scores. To the best of our knowledge, this is the first study that tests the association between RED-13 scores and task-independent low-frequency BOLD signals in healthy subjects.

## 2. Materials and Methods

### 2.1. Study Design and Participants

The current analysis was drawn from a cross-sectional study performed at the University of Medicine and Pharmacy “Iuliu Hațieganu” Cluj-Napoca, Romania between March 2022 and May 2023. Recruitment covered the metropolitan area of Cluj-Napoca and relied on social media advertising and in-person contact with the investigators. No formal sample size calculation was performed. The participants were enrolled in a larger study whose sample size was calculated for a different primary outcome: differences in resting-state brain activity according to social jetlag (data unpublished). The present analysis was therefore conducted in all eligible participants enrolled during the study period, and the sample size was determined by recruitment feasibility within the study. Prospective participants were right-handed subjects (to reduce variability related to hemispheric lateralization [21]), aged 18 to 50 years. Candidates were not eligible if they reported alcohol intake above 7 units/week (women) or 14 units/week (men), a history of diabetes, any restrictive diet at enrollment, or any psychotic disorder, anxiety, depression, or disordered eating behaviors such as anorexia, bulimia, or nocturnal eating. Additionally, those with drug abuse or recreational drug use in the 4 weeks preceding inclusion, night-shift work, travel jetlag during the previous month, pregnancy or lactation, and claustrophobia were excluded. Further exclusion criteria comprised liver transaminase > 3×UNL, eGFR < 30 mL/min/1.73 m^2^, and hyperglycemia suggestive of undiagnosed diabetes (fasting blood glucose ≥ 126 mg/dL), based on blood tests at study visit. Finally, subjects were not enrolled if they had any metallic foreign body incompatible with MRI scanning, including a pacemaker, intrauterine devices, joint prostheses and ferromagnetic osteosynthesis materials, and ferromagnetic metallic dental materials. Participants with incidental findings or with poor scan quality were additionally removed from the fMRI analysis.

We first screened potential participants through an online form that captured inclusion and exclusion criteria as reported by the participant. Those who met all inclusion criteria and none of the exclusion criteria were then invited, consecutively, to attend the study visit. Both inclusion and exclusion criteria were confirmed at the in-person study visit.

The Ethics Committee of the “Iuliu Hațieganu” University of Medicine and Pharmacy Cluj-Napoca, Romania approved the study protocol, information sheet and informed consent form (Ethics Committee approval number 23/07.02.2022). The project followed the ethical standards for research on human subjects set out in the Declaration of Helsinki of the World Medical Association, together with applicable local regulations. Before undergoing any study-related procedure, each participant received information on the purpose of the study, the procedures involved, and the collection and use of personal data, and provided written informed consent.

### 2.2. Study Assessments

During the in-person study visit, performed in the morning between 8:00 and 10:00 a.m., data on demographic parameters (age, sex), medical history, medication use, lifestyle (habitual caffeine consumption, weekly alcohol consumption, smoking status, eating behavior and average time spent being sedentary/day), sleep and appetite were collected through interviews and questionnaires. Psychiatric disorders were excluded by medical history, General Anxiety Disorder Scale (GAD-7), and Patient Health Questionnaire (PHQ9) [22,23]. Anthropometric measures (weight, height, and waist circumference) and blood pressure were obtained with calibrated scales and a sphygmomanometer. Percentage body fat was measured by bioelectrical impedance analysis using an OMRON BF511 body composition monitor (Omron Healthcare Co., Ltd., Kyoto, Japan). Blood samples were collected in fasting state (defined as an overnight fast of at least 8 h, with only water allowed) and processed the same day in the local laboratory of the Emergency Clinical County Hospital Cluj for serum aspartate and alanine aminotransferases, creatinine, and glycemia, using commercially available routine enzymatic methods (Konelab 30, Thermo Fisher Scientific Inc, Vantaa, Finland). Estimated glomerular filtration rate (eGFR) was calculated from serum creatinine using the CKD-EPI 2021 equation [24]. fMRI acquisition took place either on the same day (in 9 participants) as the study visit or on a separate day (90 participants).

Reward-driven eating was evaluated with the RED-13, a self-report instrument capturing three dimensions: preoccupation with food, loss of control over eating, and absence of satiety [15]. It was developed from the original 9-item RED [15] by adding items to extend coverage across the spectrum of reward-related eating [16]. The psychometric properties of RED-13 were established in its development study [16] and have been supported in several populations, including three European countries [19]. The Romanian version, obtained by forward–backward translation, showed excellent internal consistency in a sample of Romanian adults [25]. Its 13 items are grouped into three subscales: loss of control over eating (6 items; e.g., “When I start eating, I just can’t seem to stop”), lack of satiety (3 items), and preoccupation with food (4 items). Each item is rated on a 5-point Likert scale from 0 (strongly disagree) to 4 (strongly agree), yielding a total score between 0 and 52; subscale scores are calculated as the sum of the corresponding items. Higher total and subscale scores reflect a stronger drive to eat for pleasure and more frequent food cravings.

Subjective appetite, hunger, the amount of food one could eat, and the sensation of fullness were rated on 100 mm visual analogue scales (VASs) in response to the questions: “How hungry do you feel right now?”; “How strong is your desire to eat right now?”; “How much do you think you could eat right now?”; “How full does your stomach feel right now?”; and “How much would you enjoy eating (without regard to caloric content, fat content, or a healthy diet the following foods?”. This final question was accompanied by images of 16 food items—4 high fat low sweet, 4 high fat high sweet, 4 high sweet low fat, and 4 low fat low sweet. The VASs used were an adaptation of the Leeds questionnaire [26] and the food items used for this last question were selected based on local culture and tradition. On the 100 mm VAS, the left anchor of 0 denoted “not at all”, 50 corresponded to “moderate”, and “very much” was scored as 100 [20]. Ratings of hunger, fullness, and how much food could be eaten were analyzed directly as given by the participants. For the desire to eat the different food items, we derived composite scores for high fat, low fat, high sweet, and low sweet foods by summing the responses to the individual items.

All scales capturing food behaviors were administered to study subjects in a fasting state during the study visit. As RED-13 assesses a stable tendency toward reward-based eating rather than a transient state [15,16], its scores are not expected to depend on the time of completion or on the interval to fMRI acquisition. Similarly, resting-state metrics reflect the intrinsic functional organization of the brain rather than responses to specific eating episodes [27].

### 2.3. Resting−State fMRI

Every fMRI measurement was carried out under fasting conditions, in the 8:00 to 10:00 a.m. window. Participants were instructed to remain still with their eyes closed, to stay awake, and not to focus their thoughts on any topic during MRI acquisition.

The resting-state fMRI data was part of an experimental imaging protocol comprising a structural T1 MPRAGE sequence, a functional BOLD sequence, and diffusion-weighted imaging (DWI) scans. Acquisition used the MAGNETOM 3T Skyra (SIEMENS, Munich, Germany; 12 participants scanned) fitted with a 20-channel head coil and the Discovery MR750W 3.0 T (General Electric, Boston, MA, USA, 87 participants scanned) fitted with a 3.0 T GEM HNU head coil, as previously described [28,29]. Structural T1 MPRAGE images used an echo spacing of 6.4 ms, a bandwidth of 220 Hz/Px, 160/180 slices per volume, a TR of 1.9 s, a FOV of 230 mm, and a voxel size of 0.4 mm × 0.4 mm × 1 mm, for a total T1 acquisition time of 4 min and 22 s. Resting-state BOLD images used an echo spacing = 0.65 ms, bandwidth = 1776 Hz/Px, 160 or 180 volumes (interleaved slices/volume), slice thickness = 3 mm, matrix = 4.4 mm × 4.4 mm × 3 mm, TR = 2 s, FOV = 280 mm, and an acquisition time of 6 min and 6 s. Both structural and functional acquisitions were accelerated with GRAPPA (acceleration factor PE = 2 and ref. lines PE = 24).

A physician specialized in radiology reviewed every structural image for incidental findings.

Resting-state fMRI was selected because it reflects the intrinsic, task-independent organization of the brain, whereas responses to food cues have been reported to be more state-dependent [10]. As RED-13 was designed to capture a stable tendency toward reward-driven eating, resting-state fMRI was considered the more appropriate approach.

### 2.4. Preprocessing

Data was preprocessed with the Data Processing Assistant for Resting-State fMRI (DPARSF version 4.5) [30]. As reported previously, the pipeline began by discarding the first 10 functional volumes to permit signal equilibration, followed by slice-timing correction and spatial realignment to correct for head motion within and across fMRI runs, normalization to the Montreal Neurological Institute (MNI) space template using diffeomorphic anatomical registration through an exponentiated Lie Algebra (DARTEL) algorithm [31] and nuisance regression using the Friston 24 model including white matter and cerebrospinal fluid signals to reduce the influence of physiological noise on functional images [32,33]. Additionally, structural T1 images were co-registered to the mean functional image, segmented in grey matter, white matter, and cerebrospinal fluid and adjusted to the MNI space using the DARTEL algorithm [31]. To be included in the final analysis, a maximum of 2.0 mm and 2.0 degrees for head motion was accepted. In a further step, images or their derivates, as applicable, were smoothed with a Gaussian kernel (6 mm full-width at half-maximum [FWHM] iso-tropic) and temporally band-pass filtering was applied to retain frequencies in the 0.01 < f < 0.1 Hz band [30].

### 2.5. DC, ReHo and VMHC Calculation

First-level analysis was carried out in DPARSF, computing DC, ReHo, and VMHC from the preprocessed images. These DC, ReHo, and VMHC computations were confined to the gray matter mask produced during preprocessing. As reported previously, each individual map was converted into z-scores by dividing it by the whole-brain global mean, thereby limiting the effect of interindividual and scanner variability [34].

ReHo, DC, and VMHC characterize the brain’s functional coordination at rest. ReHo indexes the local, regional synchronization of a voxel with its immediate neighbors [35], whereas DC quantifies how many functional connections a voxel maintains with other voxels throughout the brain [36]. VMHC, in turn, reflects the functional coordination between symmetrical regions of the two hemispheres [37]. These metrics are data-driven, require no a priori selection of regions of interest, and have shown moderate to high test–retest reliability [27].

### 2.6. Statistical Analysis

No a priori sample size calculation was performed for the present analysis. All eligible participants enrolled during the study period were included. Statistical analysis was performed with IBM SPSS Statistics for Windows, Version 31.0.0.0 (Armonk, NY, USA: IBM Corp). Data are presented as mean ± standard deviation or median (quartile 1; quartile 3) for continuous variables and number (percentage) for categorical variables. The relationship of RED-13 sub-scales with VAS-derived appetite scores was assessed by partial correlation controlling for age, sex and body mass index (BMI). Statistical significance of these correlations was set at *p* < 0.0071, corresponding to a Bonferroni-corrected α of 0.05 divided by the seven comparisons performed for each RED-13 subscale.

To evaluate the relationship between the resting-state metrics and the RED-13 subscale scores, partial correlation analyses were performed using DPABI. Scanner, age, sex, and BMI were included as covariates of no interest to control for potential confounding effects (*df* = 93). Multiple comparison corrections were implemented using Gaussian Random Field (GRF) theory. The statistical maps were thresholded using a two-tailed voxel-level significance of *p* < 0.001 (corresponding to an absolute correlation threshold of *r* = 0.332), followed by a cluster-level family-wise error (FWE) correction threshold of *p* < 0.05 and were masked with the mean gray matter mask provided in DPABI. Significant clusters were further required to reach a minimum size of 18 corner-connected neighboring voxels. Each cluster was anatomically localized from the MNI coordinates of its peak voxel using the Automated Anatomical Labelling 3 [38], Brodmann [39], and Harvard–Oxford subcortical structural [40] atlases available in DPABI. Peak *r* values represent correlation coefficients, with positive values indicating positive and negative values indicating negative correlations between the RED-13 subscales and ReHo, DC, and VMHC. Correlation coefficients (*r*) are reported as measures of effect size.

## 3. Results

### 3.1. Study Sample

The study sample consisted of 103 right-handed adults fulfilling the inclusion criteria and without any exclusion criteria. Of these, one participant was excluded due to incidental finding (cavernoma) and three participants due to the presence of dental appliances that interfered with fMRI image quality. All participants had a maximum head motion ≤ 1.5 mm and ≤1.5 degree and none were excluded due to head motion.

Thus, 99 participants with complete RED-13 and fMRI data were included in the current analysis. The majority were women, with a mean age of 28.7 years and a mean BMI of 23.1 kg/m^2^. None had anxiety or depression (median GAD-7 and PHQ9 scores were both 3.0) and none consumed more than 210 mL of alcohol/week (>7 alcoholic drinks/week). For the evaluation of reward-based eating drive, the total RED-13 score was 12.0; among subscales, the lack of satiety score was 1.0, preoccupation with food was 3.0 and loss of control over eating was 9.0.

The VAS score used to assess the appetite for various food categories in fasting condition during the study visit was 300.0 for high fat foods, 440.0 for low fat foods, 410.0 for high sweet foods, and 300.0 for low sweet foods. The VAS score for hunger was 40.0, while that for fullness sensation during the visit was 10.0 (Table 1).

### 3.2. Correlation of RED-13 Subscales with VAS-Derived Appetite Scores

In the partial correlation analysis with a corrected *p*-value for significance of <0.0071, loss of control over eating and preoccupation with food subscales positively correlated with the appetite for high fat, high sweet and low sweet foods, independent of age, sex and BMI (*p* < 0.0071 for all). Additionally, loss of control over eating was positively correlated with the amount of food estimated to be eaten (partial correlation coefficient 0.323; *p* = 0.001; Table 2). None of the scales correlated with hunger or fullness sensation.

### 3.3. Correlation of RED-13 Subscales with Resting-State fMRI Metrics

**Figure 1:**
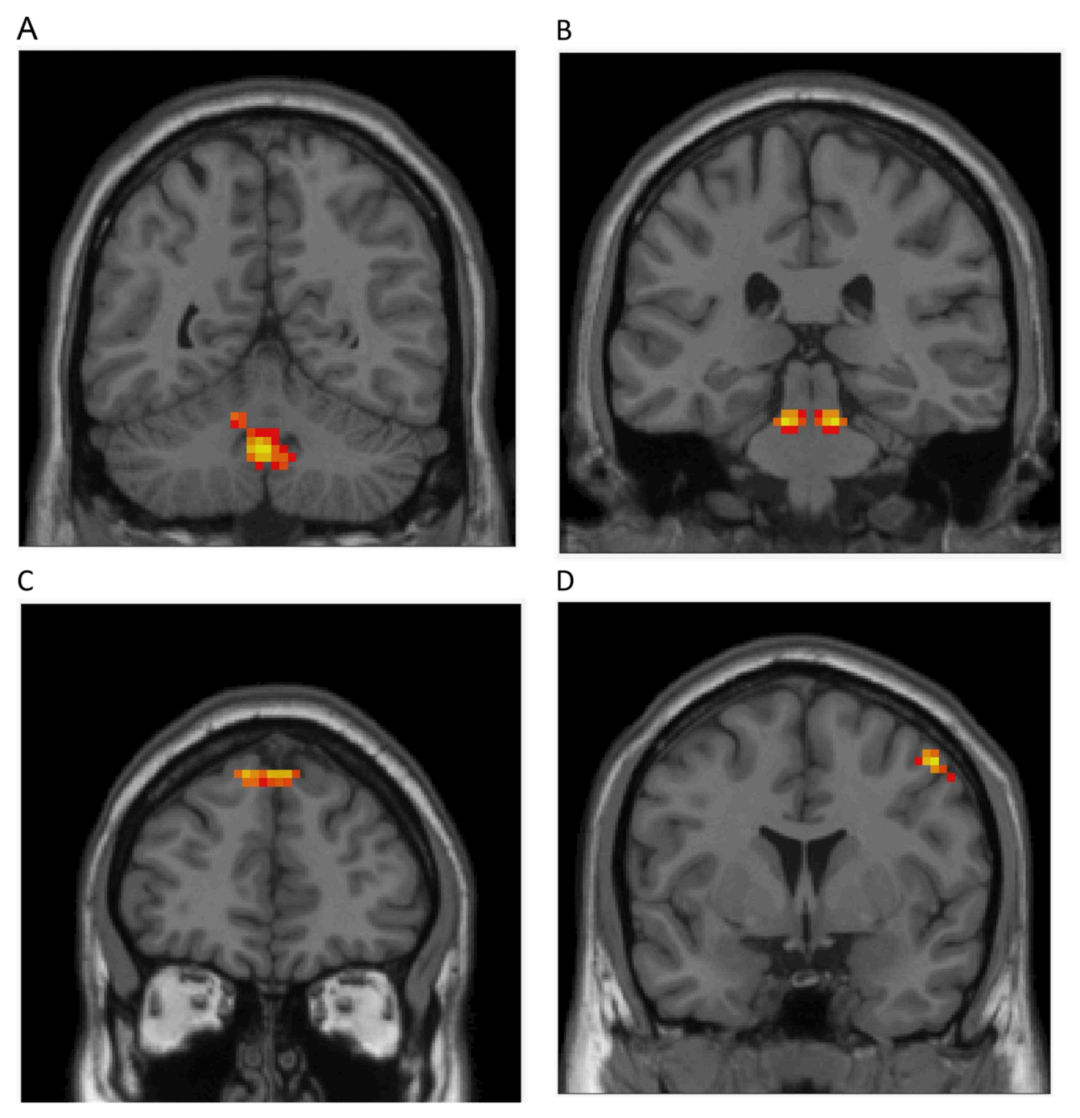
Resting-state metrics correlated with RED-13 subscales score (*n* = 99; coronal sections). (**A**) Positive correlation between RED-13 loss of control subscale with DC in left cerebellar lobule IX. (**B**) Positive correlation between RED-13 loss of control subscale and VMHC in brainstem. (**C**) Positive correlation between RED-13 lack of satiety subscale and ReHo in the left superior frontal gyrus, medial part. (**D**) Positive correlation between RED-13 preoccupation with food subscale and DC in the middle frontal gyrus. Warm colors indicate positive correlations. Peak MNI coordinates are given in Table 3. DC, degree centrality; ReHo, regional homogeneity; VMHC, voxel-mirrored homotopic connectivity; MNI, Montreal Neurological Institute.

Table 3 summarizes the correlations of RED-13 subscales with resting-state intraregional and inter-regional neuronal synchronicity and connectivity, as assessed by voxel-based ReHo, DC, and VMHC statistical maps. Loss of control over eating positively correlated with DC in the left cerebellar lobule IX (*r* = 0.416), ReHo in the left brainstem (*r* = 0.399) and negatively with the ReHo in the right calcarine sulcus, Brodmann area 19 R (*r* = −0.434). Additionally, loss of control over eating was positively correlated with the VMHC in the right and left brainstem (*r* = 0.432). The lack of satiety subscale positively correlated with the ReHo in the left superior frontal gyrus medial part (*r* = 0.389). The preoccupation with food subscale was positively correlated with DC and ReHo in the middle frontal gyrus part 2 (*r* = 0.439 and 0.394, respectively). The location of the significant selected clusters is shown in Figure 1.

## 4. Discussion

Our study’s main finding was the identification of significant associations between resting-state brain intraregional and inter-regional neuronal synchronicity and connectivity and reward-based (hedonic) eating, as assessed by RED-13 subscales. Higher scores in the RED-13 loss of control over eating subscale were correlated with higher DC in the left cerebellar lobule IX, higher VMHC between left and right brainstem, and higher ReHo in the left brainstem. Additionally, this subscale correlated with lower ReHo in the right calcarine sulcus. We also observed higher ReHo in the left superior frontal gyrus, medial segment with increased scores on RED-13 lack of satiety subscale and higher DC and ReHo in the middle frontal gyrus part 2 with increased scores on RED-13 preoccupation with food subscale.

The cerebellum has been proposed as a hub in reward-based eating; while the homeostatic drive to eat is governed by the hypothalamus, the cerebellum has been shown to modulate the brain’s mesolimbic dopamine reward system. It forms a direct functional loop with the ventral tegmental area and the nucleus accumbens and modulates dopamine release in the reward pathway. Cerebellar lobule IX is integrated into the default mode network and, alongside other non-motor cerebellar subregions, processes motivation, valuation, and learning, thereby maintaining functional coherence with the primary components of the mesolimbic reward system [41]. Through these mechanisms, cerebellar lobule IX has been involved in reward anticipation, calculation of food reward value, response to food cues, and contributes to loss of dietary control [41,42,43,44]. The higher resting-state DC in cerebellar lobule IX that we found to correlate with greater loss of control over eating is therefore consistent with this region’s integration into cognitive, affective, and homeostatic networks. Moreover, given that the DC measures how many functional connections a specific brain node has to all other regions, an increased DC of lobule IX may reflect a more connected informational hub that could deliver more food-reward information to the brain’s executive control centers [45]; however, this interpretation remains speculative.

We also found increased functional synchronization of brain signals at rest between the left and right brainstem and increased local synchronization of neurons in left brainstem, correlated with higher scores on loss of control over eating subscale. Increased ReHo in the brainstem reflects that local neurons are firing in a highly coordinated rhythm while VMHC in this region indicates increased coordination between the left and right sides of this structure [46,47]. The brainstem contains key structures of the brain’s reward and motivation pathways, including the ventral tegmental area, the substantia nigra (pars compacta), and the subventricular tegmental nucleus. Additionally, it contains the solitary tract nucleus (NTS) and parabrachial nucleus, structures involved in processing of the satiety signals (gut hormones and gastric distension) which are routed to the reward system [48,49]. Through dopamine generation, these nuclei initiate the signaling cascades that encode anticipation, wanting, and reinforcement learning. Brainstem nuclei also regulate the reward circuit by acting on higher limbic structures via serotonin, glutamate, and GABA interactions [46,50], and by relaying internal or external sensory cues to higher centers, such as the ventral striatum [46]. Thus, the increased ReHo in brainstem and VMHC between the left and right side observed in our study could be related to disrupted satiety signal, an amplification of the rewarding properties of highly palatable foods and hedonic drive to eat beyond satiety, alongside silencing of homeostatic inhibitory signals [47], although our design does not allow inferences about underlying mechanisms. Behaviorally, this association is in line with the RED-13 item “When I start eating, I just can’t seem to stop”.

Lower regional homogeneity in the right calcarine sulcus was correlated in our study with a higher loss of control over eating, and thus a perceived decrease in behavioral control over food ingestion. The calcarine cortex houses the primary visual cortex and interacts with the reward system by modifying its activity based on anticipation and conditioning of the reward visual stimuli, enhancing the attention toward the reward-predicting cue [51,52]. The calcarine cortex is connected to reward centers, and neuromodulation treatments used in addiction have been associated with reduced connection between calcarine cortex and nucleus accumbens related to reduced cravings [53]. In obesity, intermittent caloric restriction associated with successful weight loss resulted in increased ReHo in the calcarine cortex and reduced food disinhibition [14]. All these findings are consistent with the hypothesis that reduced ReHo in the calcarine cortex, as observed in our analysis, may be related to loss of conscious control over cravings through a visual cortex focused on food rewards.

Increased scores for lack of satiety were correlated in our study with an increased ReHo in left superior frontal gyrus, medial segment. This cortical region plays a role in high-level cognitive processing (working memory, cognitive flexibility, and motor planning), emotional control, and self-awareness [54]. Additionally, this cortical region is part of the default mode network and is self-referential and involved in value-related processing [55], tracking the personal relevance and subjective value of a stimulus rather than its sensory properties (“how much do I want this”). In food craving studies, this region was correlated with both increased intensity of cravings and successful downregulation of cravings by reappraisal of long-term consequences [56,57] according to the subjective valuation and regulation during food cue exposure [58]. The association observed in our study, although not related to food cravings, might reflect weak interoceptive signals of fullness sensation.

Preoccupation with food was correlated in our study with higher DC and ReHo in the right middle frontal gyrus part 2. The right middle frontal gyrus is part of the prefrontal cortex and has been implicated in cognitive control and reorienting of attention [59,60]. Its posterior region extends to BA6, a premotor region involved in motor planning and preparation of voluntary movements [61]. Neuroimaging studies have reported prefrontal activation in middle frontal gyrus in response to food cues or during the regulation of food intake [62,63]. Preoccupation with food reflects the cognitive dimension of food craving—recurrent thoughts about food [16,64]. Increased DC in this region correlated with higher scores in preoccupation for food subscale may indicate a stronger integration of this area within brain networks in individuals who report more food-related thoughts. Increased DC in the BA 6 indicates increased local synchronization of spontaneous activity in this area of the premotor cortex. Whether these associations are related to motor readiness for food-seeking behavior should be tested in future studies involving food cue paradigms.

The correlation of the loss of control over eating and preoccupation with food RED-13 subscales with an increased appetite/craving for high fat, high sweet food, as assessed by VAS and reported in a fasting condition, is in line with the observed associations with ReHo, DC and VMHC in brain regions involved in reward processing. RED-13 was designed as a stable trait metric measuring overall propensity toward reward eating, while the appetite for food assessed by VAS acts as a state metric capturing immediate, real-time anticipatory cravings. These associations are not new and have been previously reported. For example, Mason, et al. showed a strong positive correlation of RED-9 score with the Power of Food Scale, which assesses the appetite for highly palatable foods on a 5-point scale [16]. In the cross-cultural adaptation of RED-13 in three European countries, all subscales were positively correlated with craving for savory and sweet foods, as assessed by craving-related items adapted from the Control of Eating Questionnaire, with the strongest correlation observed for the loss of control over eating [19]. Loss of control captures impulsive aspects of eating behavior, which are central to reward/hedonic eating, in line with the resting-state correlates in reward-related areas observed in our sample. Since the majority of participants were of normal weight, our results are less likely to be influenced by alterations in resting-state connectivity associated with obesity [65] and may instead reflect reward-based eating behavior. Identifying such patterns before weight gain may support early prevention, which should be tested in longitudinal studies.

*Limitations and strengths*. This study has some limitations that should be acknowledged. We adopted DC, ReHo and VMHC to quantify differences in local synchronization and connectivity; however, all capture local and regional properties of brain function [66,67]. The study design focused only on resting-state fMRI and did not include a food-cue paradigm during the fMRI, instead assessing food craving before fMRI scan using VASs. Thus, the actual effect of exposure to food stimuli can only be postulated. In addition, appetite ratings and fMRI were not always performed on the same day, so VAS scores may not fully reflect the appetite state during scanning. However, both RED-13 and resting-state fMRI performed in a fasting condition are thought to reflect relatively stable, trait-like eating behavior and reward circuitry properties rather than fluctuating state variables. Additionally, the menstrual cycle phase of female participants was not recorded, although sex hormones fluctuating across the cycle may influence appetite and eating behavior [68]. The sample was a convenience sample of young, predominantly female, normal-weight and healthy participants, which may not fully represent the demographic of the general European population, potentially limiting generalizability, particularly to individuals with overweight or obesity. In addition, no sample size calculation was performed specifically for the present analysis. However, the relatively large sample size (*n* = 99 with analyzed data) for an fMRI study [69] and the restrictive inclusion and exclusion criteria, which limited sample heterogeneity, partially address this limitation. Nevertheless, this study may have been underpowered to detect small effects, and these findings should be confirmed in larger cohorts. Finally, the cross-sectional design allows only associations between RED-13 scores and resting-state metrics to be identified and does not establish causality, direction of effects or underlying mechanisms.

## 5. Conclusions

In summary, this study provides evidence that reward-based/hedonic eating, as assessed by RED-13 subscales, is associated with resting state local synchronization and regional brain connectivity within reward circuitry. Loss of control over eating was linked to a distinct pattern of increased synchronicity and connectivity in the cerebellum lobule IX and brainstem, and reduced local synchronization in the right calcarine sulcus, possibly reflecting a combination of greater subcortical reward drive and reduced cortical regulatory control. Lack of satiety, by contrast, was associated with increased local synchronization in the left superior frontal gyrus, medial segment, a region implicated in self-awareness, and regulation of food cravings based on the subjective valuation of food-related stimuli. Preoccupation with food was associated with increased DC and ReHo in the middle frontal gyrus, a region implicated in cognitive control and attentional reorienting and activated in food cue studies. Together, these findings suggest that distinct neural patterns may be associated with different dimensions of reward-based eating. These neural correlates are further supported by the association of RED-13 subscales with food craving observed in our sample, consistent with the idea that RED-13 captures a trait-like disposition toward reward-driven eating that may be reflected in the resting-state, task-independent organization of the brain’s reward and control networks, and not a state (a transient response to food cues).

Identifying resting-state patterns associated with specific aspects of reward-based eating—loss of control versus lack of satiety versus preoccupation with food—may help refine eating behavior associated with the development of overweight and obesity. Additionally, these results may contribute to the growing data that points toward a possible involvement of the reward circuitry in addition to impaired self-regulation or homeostatic dysregulation in overweight and obesity development. Future studies with longitudinal designs and more diverse and clinical populations will be needed to determine whether these resting-state patterns predict weight trajectory or treatment response.

## Figures and Tables

**Table 1:** Sample characteristics.

Variable	*n* = 99
Age, years	28.7 ± 7.9
Women, *n* (%)	65 (65.7%)
Glycemia, mg/dL	79.3 ± 8.5
ASAT, UI/L	22.0 ± 7.5
ALAT, UI/L	20.6 ± 14.6
eGFR, mL/min/1.73 m^2^	109.9 ± 13.7
Alcohol consumption per week, mL	0.0 (0.0; 28.0)
GAD-7	3.0 (1.0; 4.0)
PHQ9	3.0 (2.0; 5.0)
BMI, kg/m^2^	23.1 ± 3.7
Overweight and obesity, %	25 (25.3%)
Percentage body fat, %	28.7 ± 8.9
RED-13 score	12.0 (7.5; 20.0)
RED-13 lack of satiety	1.0 (0.0; 3.0)
RED-13 loss of control over eating	9.0 (5.0; 13.0)
RED-13 preoccupation with food	3.0 (1.0; 5.0)
VAS High fat	300.0 (150.0; 450.0)
VAS Low fat	440.0 (320.0; 550.0)
VAS High sweet	410.0 (315.0; 535.0)
VAS Low sweet	300.0 (200.0; 430.0)
Hunger	40.0 (15.0; 50.0)
How much food you can eat	50.0 (30.0; 55.0)
Fullness sensation	10.0 (0.0; 30.0)

ASAT, aspartate aminotransferase; ALAT, alanine aminotransferase; GAD-7, General Anxiety Disorder Scale; PHQ9, Patient Health Questionnaire; BMI, body mass index; RED-13, Reward-based Eating Drive Scale (13−item); VAS, visual analogue scale.

**Table 2:** Correlation of RED-13 subscales with VAS-derived appetite scores.

	Lack of Satiety r (Unadjusted *p*-Value)	Loss of Control over Eating r (Unadjusted *p*-Value)	Preoccupation with Food r (Unadjusted *p*-Value)
VAS high fat	0.263 (0.010)	**0.334 (<0.001)**	**0.314 (0.002)**
VAS low fat	0.208 (0.042)	0.264 (0.009)	**0.272 (0.007)**
VAS high sweet	0.228 (0.025)	**0.309 (0.002)**	**0.276 (0.006)**
VAS low sweet	0.254 (0.012)	**0.303 (0.003)**	**0.324 (0.001)**
Hunger	0.031 (0.766)	0.158 (0.125)	0.162 (0.116)
How much food you can eat	0.185 (0.072)	**0.323 (0.001)**	0.243 (0.017)
Fullness sensation	0.101 (0.327)	0.006 (0.952)	0.179 (0.081)

RED-13, Reward-based Eating Drive Scale (13-item); VAS, visual analogue scale. Values are correlation coefficients (*r*) with the corresponding *p*-values in parentheses; *p* < 0.05 was considered statistically significant.

**Table 3:** Brain regions significantly correlated with RED-13 subscales.

fMRI Metric	Brain Region with Clusters That Showed Significant Correlations with Subscale Score	Brodmann Area	MNI Coordinates of the Cluster			Cluster Size (Voxels)	Peak *r*
			x	y	z		
**RED-13 lack of satiety**							
DC	–						
ReHo	Superior frontal gyrus, medial segment L		3	45	48	49	0.389
VMHC	–						
**RED-13 loss of control over eating**							
DC	Cerebellar lobule IX L	–	−3	−54	−36	89	0.416
ReHo	Calcarine R	19 R	30	−63	9	39	−0.434
	Brainstem L	–	−3	−42	−30	53	0.399
VMHC	Brainstem R	–	9	−30	−24	176	0.432
	Brainstem L	–	−9	−30	−24	176	0.432
**RED-13 preoccupation with food**							
DC	Middle frontal gyrus part 2 R	6 R	48	3	51	18	0.439
ReHo	Middle frontal gyrus part 2 R	–	58	18	42	22	0.394
VMHC	–						

DC, degree centrality; ReHo, regional homogeneity; VMHC, voxel-mirrored homotopic connectivity; L, left; R, right; MNI, Montreal Neurological Institute. Coordinates are given in millimeters—indicates that no cluster reached significance. Analysis was adjusted for age, sex, body mass index and scanner.

## Data Availability

The data that support the findings of this study are available from the corresponding author, upon reasonable request.

## Abbreviations

The following abbreviations are used in this manuscript:

BMI	Body mass index
DARTEL	Diffeomorphic anatomical registration through exponentiated Lie Algebra
DC	Degree centrality
eGFR	Estimated glomerular filtration rate
FWE	Family-wise error
fMRI	Functional Magnetic Resonance Imaging
FWHM	Full-width at half-maximum
GAD-7	General Anxiety Disorder Scale
GRF	Gaussian Random Field
MNI	Montreal Neurological Institute
PHQ9	Patient Health Questionnaire
ReHo	Regional homogeneity
RED-13	Reward-Based Eating Drive Scale
UNL	Upper normal limit
VAS	Visual analogue scales
VMHC	Voxel-mirrored homotopic connectivity
